# Genetic diversity and phylogeography of *Daphnia similoides sinensis* located in the middle and lower reaches of the Yangtze River

**DOI:** 10.1002/ece3.4880

**Published:** 2019-04-04

**Authors:** Jianxun Wu, Wenping Wang, Daogui Deng, Kun Zhang, Shuixiu Peng, Xiaoxue Xu, Yanan Zhang, Zhongze Zhou

**Affiliations:** ^1^ Anhui Key Laboratory of Resource and Plant Biology, School of Life Science Huaibei Normal University Huaibei China; ^2^ School of Resource and Environmental Engineering Anhui University Hefei China

**Keywords:** *Daphnia similoides sinensis*, genetic diversity, molecular marker, phylogeography, The Yangtze River

## Abstract

Geographical patterns, climate, and environmental change have important influences on the distribution and spread of aquatic organisms. However, the relationships between the geographical pattern and phylogenetics of *Daphnia* as well as environmental change are not well known. The genetic diversity and phylogeography of seven *D. similoides sinensis* populations located in the middle and lower reaches of the Yangtze River were investigated based on the combination of mitochondrial (*CO*I gene) and nuclear (14 microsatellite primers) markers. Based on the mitochondrial gene markers, *D. similoides sinensis* from the middle and lower reaches of the Yangtze River had one ancestral haplotype and two evolutionary clades. In addition, *D. similoides sinensis* population deviated from neutral evolution, showing signs of a bottleneck effect followed by population expansion. Based on the microsatellite markers, the seven *D. similoides sinensis* populations formed three main groups. The dendrogram (NJ/ME) showed that *D. similoides sinensis* based on the mitochondrial genes marker were obviously clustered two main clades, whereas there were three clades based on the microsatellite markers. Our results suggested that the habitat fragmentation due to the barrier of the dams and sluices promoted the genetic differentiation and phylogeography of *D. similoides sinensis* populations in the middle and lower reaches of the Yangtze River.

## 1 INTRODUCTION

Geographical patterns, climate, and environmental changes have important influences on the genetic composition, population distribution, and species diversity of aquatic organisms (Hewitt, 2000; Petit et al., 2003). Avise et al. (1987) presented the concept of intraspecific phylogeography, whose basic principle was to study the relationship between gene genealogy and geography of organisms. The genealogical analysis and temporal–spatial distribution of haplotypes could be used to estimate the historical process of species differentiation between closely related species or at the intraspecific level (Avise, 1998; Zuykova, Bochkarev, & Sheveleva, 2016).

The phylogeography of organisms can be effectively revealed using multiple molecular markers. By using the mitochondrial *CO*I and NDI genes, Ober, Matthews, Ferrieri, and Kuhn (2011) found that most mountain ranges resulted in the population differentiation of *Scaphinotus petersi* distributed on Sky Islands in southeastern Arizona during the postglacial maximum times. Based on the *16S* rDNA, *CO*I gene, and *18S* rDNA molecular markers, Wang et al. (2016) concluded that the phylogenetics of the cladoceran *Daphnia pulex* located in ten habitats of the middle and lower reaches of the Yangtze River was related to its geographical location. In the nuclear genome, microsatellite markers have widely been applied to phylogeography because of their high polymorphism, stability, codominance, and Mendelian inheritance (Lane, Symonds, & Ritchie, 2016; Mobley, Small, Jue, & Jones, 2010).

**Figure 1:**
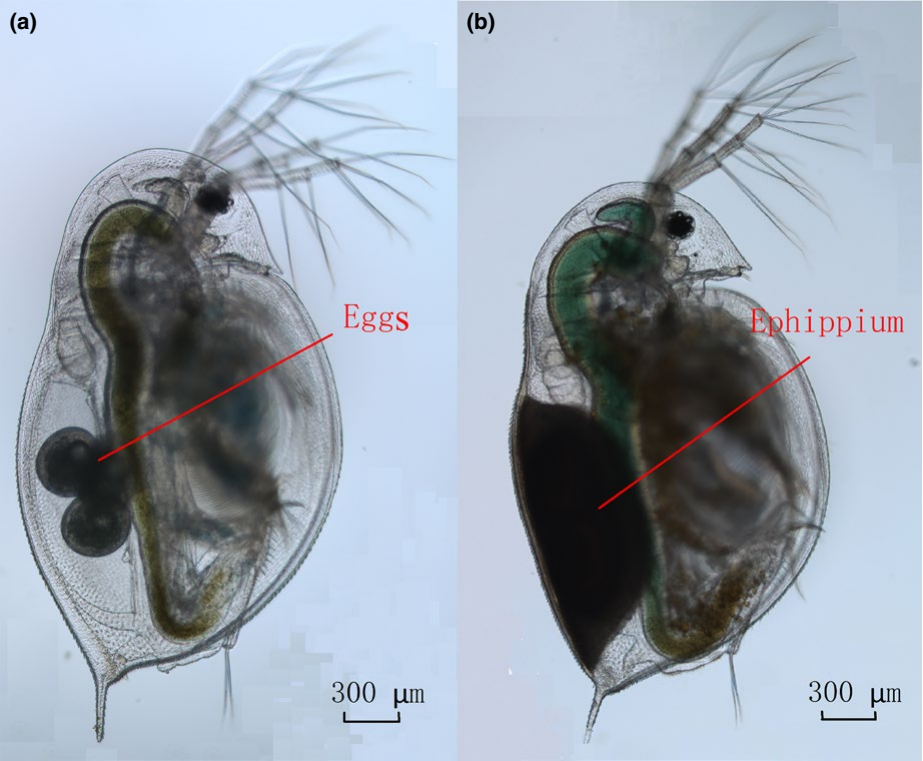
The photograph of *Daphnia similoides sinensis *female (a: parthenogenetic female, b: sexual female)

Zooplankters are an important part of aquatic food chains and have important ecological roles in aquatic ecosystems. *Daphnia* is a common crustacean zooplankton, having the characteristics of wide distribution, rapid reproduction, and sensitivity to environmental changes (Su, 2013). So, *Daphnia* is often regarded as a model organism for the study of bio‐toxicology, genetics, and ecology (Hebert, 1978; Lampert, 2011). Moreover, *Daphnia* has a weak swimming ability because of a small body size (Rand, 1996). *D. similoides sinensis* is distributed in eutrophic ponds and lakes in Southern Asia, from Pakistan to Indonesia, and China (Benzie, 2005). *D. similoides sinensis* perform cyclic parthenogenesis under good conditions, whereas they convert to sexual reproduction and produce resting eggs when environmental conditions worsen, such as low temperature, large predation pressure, or high population density (Figure 1). This species was previously recorded as *D. similis* or *D. carinata* in China (Gu, Xu, Lin, Henri, & Han, 2013; Jiang & Du, 1979; Xu et al., 2014). *D. similoides sinensis* was observed in some lakes located in the middle and lower reaches of the Yangtze River, China (Chen, Chen, Li, & Zhao, 2009; Ma et al., 2016), which coexisted with *Daphnia pulex* and *Daphnia galeata* (Deng et al., 2008).

**Figure 2:**
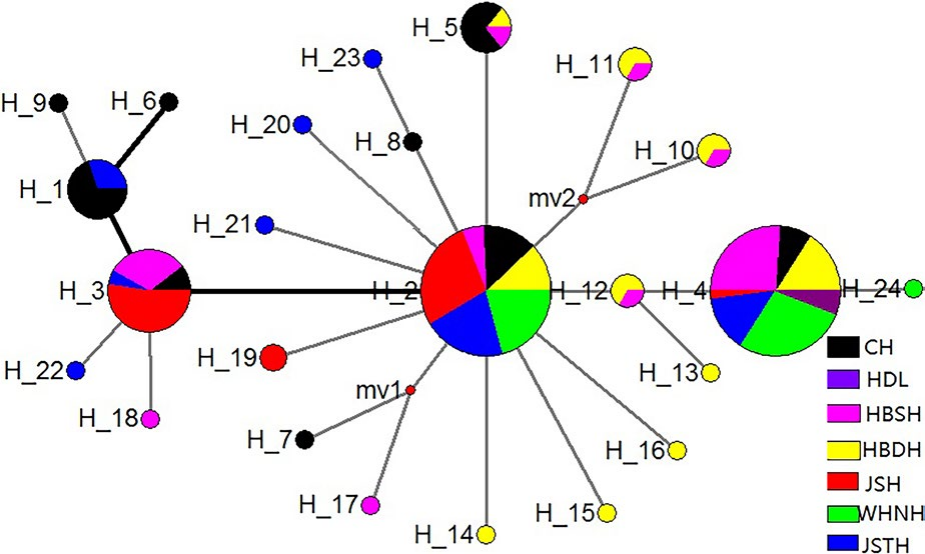
Networks of haplotype of seven *Daphnia similoides sinensis* populations located in the middle and lower reaches of the Yangtze River. The haplotypes are showed by different colors based on seven populations. The size of circle stands for the number of haplotypes

The Huai River historically drained directly into the Yellow Sea, but it is now connected to the lower reaches of the Yangtze River after many floods. Along the middle and lower reaches of the Yangtze River and Huai River of China, many tributaries of the river and lakes are distributed (Figure 2). There were nineteen floods in the middle and lower reaches of the Yangtze River from 1921 to 2000 (Shi, Jiang, Su, Chen, & Qin, 2004). Many dam and sluice projects in the region have been built since 1950s in order to store water or prevent the flooding, and some lakes have changed from a natural type into a reservoir type (Wang & Dou, 1998). In Lake Chaohu, the lake was isolated from the Yangtze River due to the construction of Chaohu dam and Yuxi dam in the 1950s. Similarly, the connection of Lake Junshan with Lake Poyang and the Yangtze River was cut off after the construction of the lake embankment in 1958 (Wang & Dou, 1998). In Wuhan city, Lake Nanhu had become a closed lake as a result of the development of the city. The building of dams and sluices can form a barrier for the migration and communication of aquatic organisms (Naiman, Melillo, Lock, Ford, & Reice, 1987; Yi, Yang, & Zhang, 2010), resulting in changes in species diversity or genetic diversity. About 28 species of fish have disappeared since 1950 in Lake Zhangdu due to the construction of artificial barriers between the rivers and lakes (Wang, Hu, & Wang, 2005). In the Three Gorges reservoir area of the Yangtze River, seven *Leiocassis longirostris *populations diverged into two groups after the construction of the Three Gorges Dam (Li, 2007). Moreover, natural linkage between the Huai River system and the Yangtze River system was also isolated after the construction of Sanhe sluice in 1953. How these changes in hydrology influence the genetic diversity and phylogeography of aquatic organisms (e.g., *Daphnia*) located in the middle and lower reaches of the Yangtze and Huai Rivers is not yet clear.

In this study, both mitochondrial (*CO*I gene) and nuclear (14 microsatellite primers) markers were jointly used to study the genetic diversity of *D. similoides sinensis*. This study aims to compare the differences in phylogenetics and population genetics of *D. similoides sinensis* located in the middle and lower reaches of the Yangtze River and to explore the influence of the geographical pattern on the phylogenetics of *D. similoides sinensis*. Specifically, we have made a hypothesis that the construction of dam and sluices in the middle and lower reaches of the Yangtze River promoted the genetic differentiation of *D. similoides sinensis*.

## 2 MATERIALS AND METHODS

### 2.1 Animal culture and DNA extraction

*Daphnia similoides sinensis *was collected from water bodies located in the middle and lower reaches of the Yangtze River, belonging to water systems of the Yangtze River and Huai River (Table 1). In the laboratory, animals were identified (Benzie, 2005; Jiang & Du, 1979) and then monoclonally cultured in an intelligent light incubator (Ningbo Saifu, China) with the illumination of 12 hr light: 12 hr dark at (25 ± 1)°C. *Scenedesmus obliquus* was used as their food. Before extracting complete DNA of monoclonal *D. similoides sinensis*, each adult individual was drawn by a straw and rinsed with double‐distilled water, and then crushed with a sterile 10 μl pipette tip. Genomic DNA of *D. similoides sinensis* was extracted by the TIANamp Micro DNA Kit (Tiangen, Beijing).

**Table 1:** Origin and number of *Daphnia similoides sinensis*

Sampling locations	Population code	Coordinates		No. individuals
Lake Nanhu, a lake in Hubei Province	WHNH	E114.367663°	N30.485492°	27
Lake Junshan, a lake in Jiangxi Province	JSH	E116.323442°	N28.549058°	29
Lake Chaohu, a lake in Anhui Province	CH	E117.377343°	N31.648290°	30
Lake Taihu, a lake in Jiangsu Province	JSTH	E120.212168°	N31.411620°	27
A pond in East China Normal University, Shanghai	HDL	E121.401500°	N31.228696°	3
Dai River, a river in Anhui Province	HBDH	E116.847682°	N33.957697°	28
Sui River, a river in Anhui Province	HBSH	E116.784559°	N33.911004°	28

### 2.2 PCR amplification

Fourteen pairs of primers were used for the microsatellite markers (Table 2). The *CO*I gene was amplified with the LCO1490 (5′‐GGTCAACAAATCATAAAGATATTGG‐3′) and HCO2198 (5′‐TAAACTTCAGGGTGACCAAAAAATCA‐3′) (Xu et al., 2014).

**Table 2:** Microsatellite marker primers employed in this study

NCBI code	Primer（5′‐3′）	SSR	Fluorescent mark type
KY440958	AACACAGAACTACCTGGCGG	(TC)10	5′‐FAM
	GAAAAGGGACAGGTGAGGGG		
KY440961	AGCGGCTTCCAATCTACGTC	(GT)10	5′‐HEX
	GAGTTACCGCACATAGCCGA		
KY440963	AGGAAGCGAACTGGAACACA	(AC)10	5′‐FAM
	TCCAAATTCGGTCGAGGGTT		
KY440966	CACACGCGCATAACTCGAAA	(GT)10	5′‐FAM
	GGCCGGTGACACGATGATAT		
KY440964	CCCGTTGTCCCTGTCTCTTC	(CA)10	5′‐HEX
	CACGTGGAGTCTTGGTGTGA		
KY440968	CCCTGGATCAAAGCGGAAGA	(TC)10	5′‐HEX
	CCGAGGCCTTGTGTGTACAT		
KY440960	GGAACGTAACCCCTAGCGTC	(CA)10	5′‐FAM
	GCGATGTAATTTGCGGGCAA		
KY440965	ACAAGGAGAGGCCAACGATG	(GGA)5	5′‐HEX
	CCCAAGTCACCTTAAACCCGA		
KY440959	ACACTGGGCTGCAAAGTCTT	(TCT)5	5′‐FAM
	CCTTCGTTCGTGTATGCCCT		
KY440962	ACAGCAGCCGATGAAAGTCA	(CAA)5	5′‐FAM
	TGTTGTTGCTGTTGCTGGTG		
KY440967	ACAGGAGAAGTCCAAGTGCG	(TCA)5	5′‐HEX
	ATGAAAGTGGGTCACGGTGC		
AF233360	ACGCGTTTCATCCTGACCC	(AC)8	5′‐HEX
	GCCTTGTTGTTTCTTGCCTC		
AF233362	GGGAAATAAAGAAGAACCGC	(AC)9	5′‐HEX
	ACAGCTAACACAAGTTGATAC		
AY057865	AGTCGCGACGACATAAAGC	(TG)6(GA)7	5′‐FAM
	GTGGTAGTTGTGGAATCCG		

The PCR system (25 µl) of the *CO*I gene contained 1.0 µl of genomic DNA (100 ng/μl), 2.5 µl of 10 × LA‐Taq Buffer II, 4.0 µl of dNTPs (2.5 mM) (Shanghai Shenggong, China), 0.5 µl of MgCl_2_ (25 mM), 1.0 µl of each primer (10 mM) (Shanghai Shenggong, China), 0.25 μl of DNA polymerase TaKaRa‐LA‐Taq (5 U/µl) (Clontech, USA), and 14.75 µl of double‐distilled H_2_O. The PCR system (25 µl) of the SSRs contained 1.0 µl of genomic DNA (100 ng/μl), 1.0 µl of each primer (10 mM) (Shanghai Shenggong, China), 12.5 µl of 2 × Taq Master Mix (BioTeke Corporation, China), and 9.5 µl of double‐distilled H_2_O.

The conditions of the *CO*I gene amplification included an initial denaturing step of 1 min at 95°C, 35 cycles of 40 s at 95°C, 40 s at 45°C, and 1 min at 72°C, as well as a final extension of 72°C for 10 min. The conditions of the SSR amplification included an initial denaturing step of 3 min at 95°C, 35 cycles of 45 s at 95°C, 45 s at 54°C, and 45 s at 72°C, as well as a final extension of 72°C for 10 min.

### 2.3 Electrophoresis, sequencing, and data analyses

The PCR amplification products of the *CO*I gene were checked by gel electrophoresis and then purified by the AxyPrep DNA Gel Recovery Kit (AxyPrep, USA) and sequenced with the forward and reverse primers (GenScript, Nanjing). The sequence alignment was carried out using the SeqMan software in DNAStar. DNAspV5 was used to analyze the site variations, haplotype diversity, and nucleotide diversity of *D. similoides sinensis* among the *CO*I sequences, as well as *F*_st_ among populations. *Fu's Fs* test, *Tajima's *neutrality test, and mismatch distribution were used to detect the evolutionary history of *D. similoides sinensis *populations (Fu, 1997; Tajima, 1989) using DnaSP Version 5. The dendrogram (NJ/MP) of seven *D. similoides sinensis* populations based on the *F*_st_ values was constructed with MEGA 4.1. The genetic distances of seven *D. similoides sinensis* populations were calculated using MEGA 4.1. The genetic distances among sequences were calculated by the Kimura's two‐parameter model with 1,000 bootstraps. Phylogenetic tree of *Daphnia* individuals based on Maximum likelihood (ML) estimates was constructed with MEGA 4.1 and bootstrap resampled 1,000 times.

The *D. similoides sinensis* samples from seven populations located in the middle and lower reaches of the Yangtze River were amplified using the 14 microsatellite fluorescent labeled primers (Table 2). The PCR products were checked by both agarose gel electrophoresis and capillary electrophoresis using an ABI 3730 sequencer. The number of observed and effective alleles, expected heterozygosity, shannon information index and polymorphic ratio were calculated using the Popgene version 1.31 software (Yeh, Yang, & Boyle, 1999), as well as the genetic distances among populations. Based on the microsatellite markers, *F*_st_ among populations were performed by Arlequin 3.5 software to analyze the differences in the genetic diversity among seven *D. similoides sinensis* populations. The population genetic structure of *D. similoides sinensis* was reconstructed using the 14 polymorphic microsatellite loci in Structure 2.3.1. Posterior likelihood values were calculated from *K* = 1 to *K* = 10 using the LOCPRIOR model (Hubisz, Falush, Stephens, & Pritchard, 2009). For each *K*, 10 simulations with a burn‐in of 10,000 Markov Chain Monte Carlo (MCMC) iterations were run, and then 100,000 iterations after burn‐in were performed. The most likely value for *K* was estimated from the greatest rate of change in the likelihood function (Δ*K*) of successive *K* values (Evanno, Regnaut, & Goudet, 2005; Frisch et al., 2017). Mantel's tests were used to measure the geographical distances and genetic distances (*F*_st_) among seven geographical populations of *D. similoides sinensis* with tools for population genetic analyses (TFPGA) version 1.3 (Miller, 1997).

## 3 RESULTS

### 3.1 Analysis on the genetic diversity and haplotype structures of seven *D. similoides sinensis* populations located in the middle and lower reaches of the Yangtze River based on the *CO*I gene marker

Among seven *D. similoides sinensis* populations, 172 mitochondrial gene sequences were obtained, and the number of identified base sites was 478. The nucleotide diversity (*π*) and haplotype diversities (Hd) were, respectively, 0.0053 and 0.784. A total of 24 haplotypes were detected, including 8 shared haplotypes and 16 specific haplotypes. The haplotype networks indicated that seven *D. similoides sinensis* populations had one ancestral haplotype (H‐2) and two main clades (Figure 2), which showed the specificity of geographical distributions. The specific haplotypes were mainly distributed in HBDH and JSTH populations, which are located in the upper reaches of the Huai River tributary and lower reaches of the Yangtze River, respectively. In addition, both JSH population (H‐2, H‐3, and H‐19) and WHNH population (H‐2, H‐4, and H‐24) were mainly composed of three haplotypes, which had significant differences from the other five populations.

### 3.2 Genetic diversity and genetic structure of seven *D. similoides sinensis* populations located in the middle and lower reaches of the Yangtze River based on the 14 SSR markers

Among seven *D. similoides sinensis* populations, the ranges of observed alleles, effective alleles, and shannon information index were 1.094–1.648, 1.063–1.185, and 0.053–0.195, respectively. The ranges of observed heterozygosity and expected heterozygosity were 0.383–0.611 and 0.471–0.692, respectively. The haplotype diversity (Hd) and nucleotide diversity (*π*) ranged from 0.553 to 0.936 and from 0.0014 to 0.0092, respectively (Table 3).

**Table 3:** Summary of genetic variation in seven *Daphnia similoides sinensis* populations

	Hd	*π*	Tajima's *D*	Fu and Li's *D*	na	ne	*h*	*I*	*P*	Exp_Het	Obs_Het
CH	0.936	0.0054	−0.683	−1.149	1.604	1.185	1.119	0.194	60.430	0.663	0.500
HBDH	0.871	0.0092	−1.176	−0.534	1.432	1.155	0.100	0.160	43.170	0.572	0.383
HBSH	0.751	0.0082	−1.243	−2.242	1.453	1.177	0.112	0.177	45.320	0.608	0.485
JSH	0.591	0.0014	−0.711	−0.951	1.648	1.170	0.115	0.195	64.750	0.692	0.438
JSTH	0.801	0.0074	−2.087*	−3.673*	1.152	1.173	0.110	0.178	51.080	0.630	0.516
WHNH	0.553	0.0022	1.002	−0.229	1.432	1.151	0.095	0.151	43.170	0.531	0.338
HDL	—	—	—	—	1.094	1.063	0.036	0.053	9.350	0.471	0.611

Notes

Exp_Het: expected heterozygosity; *h*: Nei's gene diversity; Hd: haplotype diversity; *I*: Shannon's diversity index; na: the observed number of alleles; ne: the effective number of alleles; Obs_Het: observed heterozygosity; *P*: percentage of polymorphic loci; *π*: nucleotide diversity.

* Significance at the 5% level.

Based on the microsatellite markers, the molecular variances were 4.305 within populations and 0.561 among populations, suggesting that the genetic variation in *D. similoides sinensis* located in the middle and lower reaches of the Yangtze River occurred mostly within populations. The Nei's genetic distances among seven *D. similoides sinensis* populations ranged from 0.002 to 0.008 (Table 4). The maximum genetic distance appeared between the HBDH and JSH populations, whereas the minimum was between the WHNH and JSTH populations.

**Table 4:** Geographical distances (km/above diagonal) and Nei's genetic distance (below diagonal) matrix among seven *Daphnia similoides sinensis* populations based on the SSR marker

	CH	HBDH	HBSH	HDL	JSH	JSTH	WHNH
CH		273	277	353	255	324	379
HBDH	0.007		8.5	601	423	451	526
HBSH	0.006	0.007		602	430	446	534
HDL	0.003	0.006	0.005		484	282	580
JSH	0.006	0.008	0.007	0.004		565	122
JSTH	0.005	0.006	0.004	0.003	0.005		686
WHNH	0.006	0.006	0.004	0.005	0.006	0.002	

**Figure 3:**
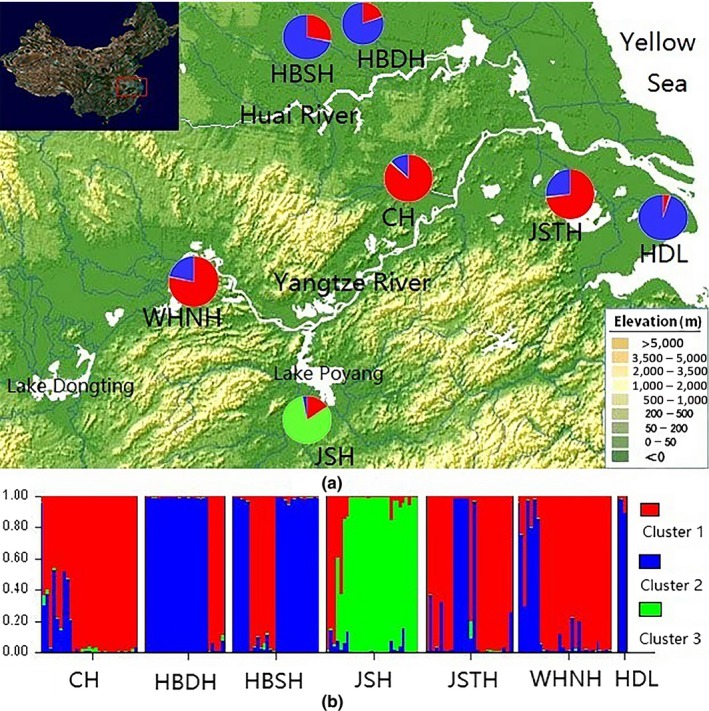
Cluster analysis of *Daphnia similoides sinensis *population structure based on the SSR markers. (a) Map of *D. similoides sinensis *population showing the proportion of each cluster among each population. (b) Proportion of each individual genome assigned to three clusters. Each individual is represented by a vertical bar

Seven *D. similoides sinensis* populations located in the middle and lower reaches of the Yangtze River were grouped into three clusters (Figure 3). Among them, cluster 1 dominated in the WHNH population, CH population, and JSTH population, and cluster 3 dominated in the JSH population, which is distributed in the Yangtze River basin. However, cluster 2 dominated in the HBSH population and HBDH population which distributed in the Huai River basin, as well as in the HDL population which located in the lower reaches of the Yangtze river.

### 3.3 Genetic differentiation of seven *D. similoides sinensis* populations located in the middle and lower reaches of the Yangtze River

Based on the microsatellite markers, there was lower *F*_st_ between the HBSH population and other six populations (Table 5), especially between the HBSH population and HBDH population (0.027). The *F*_st_ value between the JSH population and other six populations was very high (Table 5), implying that there was genetic isolation between the JSH population and other six populations. At the same time, based on the mitochondrial marker, the *F*_st_ values between the JSH population and other six populations were also higher (Table 5). The *F*_st_ values based on the mitochondrial marker were not significantly correlated with the geographical distances among populations (*r *= −0.3394, *p* = 0.735).

**Table 5:** *F*_st_ of the genetic differentiation matrix based on the SSR markers (below diagonal) and the *CO*I gene marker (above diagonal) among seven *Daphnia similoides sinensis* populations

	CH	HBDH	HBSH	JSH	JSTH	WHNH	HDL
CH		0.117	0.105	0.066	0.023	0.257	0.617
HBDH	0.148		0.010	0.152	0.032	0.054	0.311
HBSH	0.11	0.027		0.173	0.022	0.019	0.282
JSH	0.145	0.242	0.207		0.054	0.409	0.842
JSTH	0.074	0.109	0.066	0.181		0.079	0.416
WHNH	0.134	0.109	0.071	0.218	0.045		0.406
HDL	0.129	0.155	0.125	0.210	0.082	0.141	

### 3.4 Phylogeography of seven *D. similoides sinensis* populations located in the middle and lower reaches of the Yangtze River

**Figure 4:**
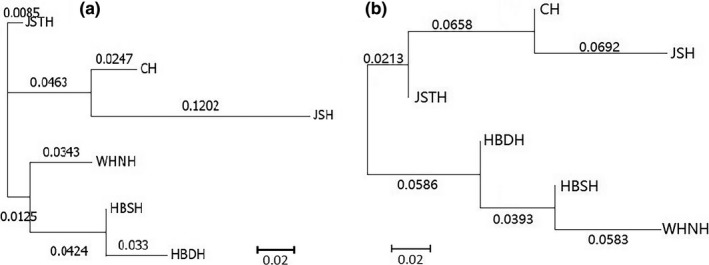
Dendrogram of six *Daphnia similoides sinensis* populations located in the middle and lower reaches of the Yangtze River. (a) Dendrogram (NJ/ME) of six *D. similoides sinensis* populations based on the SSR markers. (b) Dendrogram (NJ/ME) of six *D. similoides sinensis* populations based on the *CO*I gene marker. The numbers stand for the genetic distance between populations.

**Figure 5:**
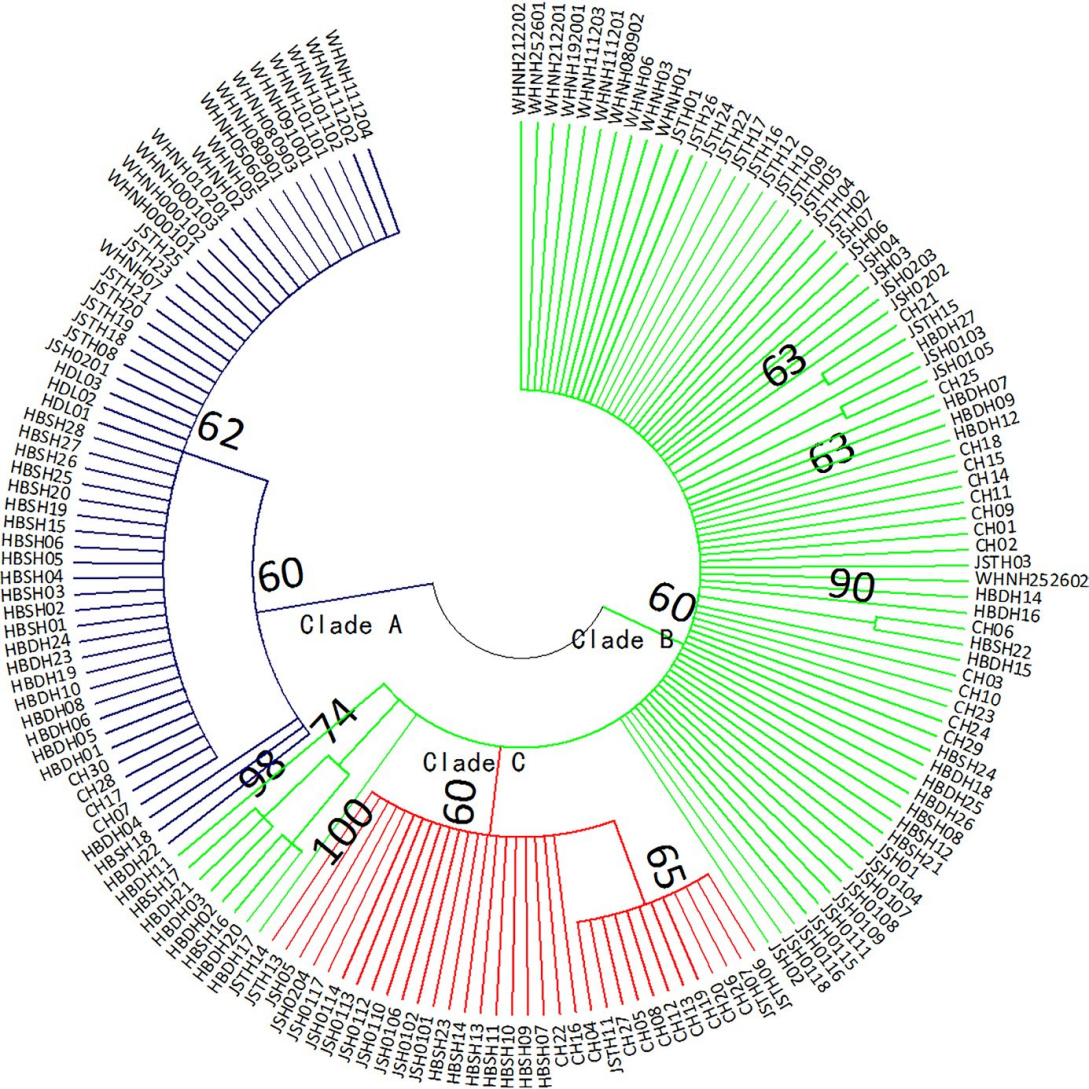
Maximum likelihood (ML) tree based on the *CO*I gene sequences of *Daphnia similoides sinensis* individuals. Bootstrap values >60% are indicated. The blue lines stand for clade A, green lines stand for clade B, and red lines stand for clade C

Because of the lower sample size of the HDL population, phylogenetic trees (NJ/ME) of only six populations (CH, HBDH, HBSH, JSH, JSTH, and WHNH population) were constructed based on both mitochondrial and microsatellite markers. Based on the microsatellite markers, the dendrogram (NJ/ME) showed that six *D. similoides sinensis* populations were obviously divided into three clades (Figure 4a). In the lower reaches of the Yangtze River, the JSTH population located in a separate clade, whereas the CH population and JSH population were clustered into another clade. The WHNH, HBSH, and HBDH populations were clustered into the third clade, among which the WHNH population is distributed in the middle reaches of the Yangtze River and the HBDH and HBSH populations in the upper reaches of the Huai River tributary. Based on the mitochondrial genes marker, the dendrogram (NJ/ME) indicated that six *D. similoides sinensis* populations were clustered into two main clades (Figure 4b). The JSH, CH, and JSTH populations were clustered into a clade, whereas the WHNH, HBDH, and HBSH populations were in the other clade. According to the mitochondrial and microsatellite markers, it was obvious that there were two evolutionary directions of *D. similoides sinensis* populations located in the middle and lower reaches of the Yangtze River. Moreover, the phylogenetic tree (ML) of *D. similoides sinensis* individuals showed also two major evolutionary clades (clade A and clade B) (Figure 5). Clade A mainly included the individuals of HBDH, HBSH, and WHNH populations, whereas clade B mainly contained the individuals of JSH, CH, and JSTH populations.

**Figure 6:**
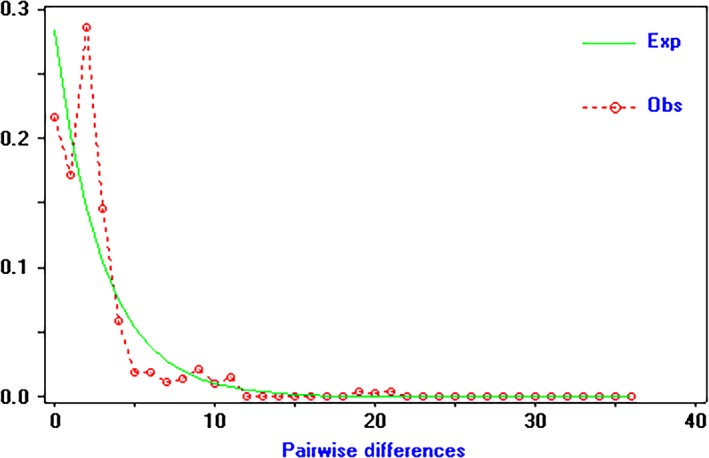
The observed pairwise difference (red line) and the expected mismatch distributions under the sudden expansion model (green line) based on the mitochondrial gene sequences of *Daphnia similoides sinensis* populations located in the middle and lower reaches of the Yangtze River

Based on the mitochondrial gene sequences, the distribution of base mismatches indicated that there was a single peak, implying that the *D. similoides sinensis* population located in the middle and lower reaches of the Yangtze River experienced an expansion process (Figure 6). Both *Fu's Fs *neutral test (*D* = −3.673, *p* < 0.02) and *Tajima's* test (*D* = −2.087, *p* < 0.05) showed that the JSTH population deviated from neutral evolution, whereas the other six populations did not deviate from neutral evolution.

## 4 DISCUSSION

### 4.1 Genetic diversity and genetic structure of *D. similoides sinensis* located in the middle and lower reaches of the Yangtze River

Haplotype diversity (Hd) and nucleotide diversity (*π*) are two important parameters to study population genetic diversity of organisms (Tajima, 1983; Weir, 1990). Higher Hd and lower *π* values in the natural population means that the organism could expand after a period of lower population size and enhance the retention of new mutations (Crandall, Sbrocco, Deboer, Barber, & Carpenter, 2011; Grant & Bowen, 1998). In this study, based on mitochondrial *CO*I gene sequences, higher Hd and lower *π* values suggested that *D. similoides sinensis* located in the middle and lower reaches of the Yangtz River experienced a bottleneck in the process of population formation. This phenomenon may be related to the rapid expansion of aquatic animal populations after the bottleneck effect and the quick accumulation of Hd, as well as periodic flooding events that occur in this region (Xu, Yu, & Ma, 2005). The *Fu's Fs* neutral test and *Tajim's *test suggested that the *D. similoides sinensis* JSTH population had experienced a bottleneck effect in the history. The JSTH population is located in Lake Taihu, which is part of the lower reaches of the Yangtze River. To improve water quality, two water transfer projects from the Yangtze River to Lake Taihu were conducted from 2002 to 2003 (Hu, Zhai, Zhu, & Han, 2008). However, the ecosystem in Lake Taihu became unstable after water transfers (Zhai, Hu, & Zhu, 2010). These water transfer projects might have resulted in the JSTH bottleneck and affected the population structure of *D. similoides sinensis* in Lake Taihu.

In this study, seven *D. similoides sinensis* populations located in the middle and lower reaches of the Yangtze River were grouped into three clusters based on the microsatellite markers. According to the location of these populations, three clusters appeared to be related to geography, which cluster 2 was dominant in the Huai River basin whereas cluster 1 dominated along of the Yangtze River. Moreover, the dendrogram (NJ/ME) based on the mitochondrial genes marker showed that six *D. similoides sinensis* populations were obviously clustered into two main clades, whereas there were three clades based on the microsatellite markers. One reason for the differing results between the mitochondria and nuclear data is significant differences in the evolutionary rate and level of polymorphism between different molecular markers (Bai & Zhang, 2014). The mitochondrial DNA have the characteristics of maternal inheritance (Avise et al., 1987), whereas the nuclear genes have higher mutation rates and are more appropriate for determining the genetic differences in organisms among different geographic populations (Al‐Hamidhi et al., 2015; Selkoe & Toonen, 2006). The above results suggested that *D. similoides sinensis *located in the middle and lower reaches of the Yangtze River show significant genetic differentiation.

### 4.2 Influence of geographic isolation on the phylogeography of *D. similoides sinensis* located in the middle and lower reaches of the Yangtze River

The influences of geographic isolation on the phylogeography of aquatic organisms have extensively been researched in the world. Machordom and Doadrio (2001) found that the geographic isolation caused the interruption of gene flow of *Luciobarbus* and then affected the phylogenetic differentiation and geographical distribution of *Luciobarbus *population. Slechtova, Bohlen, Freyhof, Persat, and Delmastro (2004) thought that the geographical isolation of the Alps Mountains resulted in the speciation of many endemic species in the Italian peninsula. In Europe, De Gelas and De Meester (2005) found that the genetic divergence of *Daphnia magna* had a high degree of provincialism with a patchy occurrence of specific lineages. Based on the mtDNA gene sequences, several *Daphnia* species in North America showed the similar phylogeographical pattern that topographic barriers generated population divergence of *Daphnia* (Hebert, Witt, & Adamowics, 2003; Penton, Hebert, & Crease, 2004; Taylor, Finston, & Hebert, 1998). In the middle and lower reaches of the Yangtze River of China, the linkage between the Huai River and Yangtze River was changed in 1953 after the construction of Sanhe sluice, and the habitats of *D. similoides sinensis* along the two rivers was also isolated. Similarly, the connection of Lake Junshan with Lake Poyang and the Yangtze River was cut off after the construction of the lake embankment in 1958. In our study, there were two evolutionary clades of *D. similoides sinensis* located in the middle and lower reaches of the Yangtze River, namely the Yangtze River clade and the Huai River clade. Based on the genetic structure of the population, further analysis revealed that the JSH population had significant differences from the other four populations in the Yangtze River clade. The Lake Junshan was interconnected with Lake Poyang before 1958, but it was separated from Lake Poyang after the construction of an embankment in 1958. The lake ecosystem changed from an open to closed status, which weakened or even interrupted gene flow between the lake and other geographical populations of *D. similoides sinensis*. Therefore, the geographic isolation affected the phylogeography of *D. similoides sinensis* located in the middle and lower reaches of the Yangtze River.

Avise (1989) argued that the phylogeny of biogeographic patterns depends on the relationship between the phylogeny and geographical distribution of population. Population differentiation of organisms tends to be closely related to geographical distances, but recent population expansion and habitat fragmentation (Templeton, Routman, & Phillips, 1995) might affect the genealogy of species. Based on an experimental platform, Golestani, Gras, and Cristescu (2012) observed a direct and continuous increase in the speed of evolution with the increasing number of obstacles in the environment, and the reduced population size could result in more pronounced genetic drift and rapid differentiation between populations. Vanoverbeke, De, and De (2007) found also that *Daphnia magna* populations from small water bodies showed a stronger among‐population genetic differentiation than the populations inhabiting larger water bodies. In the study, the Fst values based on the mitochondrial marker were not significantly correlated with the geographical distance among populations, but long‐term habitat fragmentation caused by dams and sluices could restrict the movement among the *D. similoides sinensis* populations after the 1950s and enhance the genetic differentiations of *D. similoides sinensis* populations located in the middle and lower reaches of the Yangtze River.

In conclusion, there is significant genetic structure of the *D. similoides sinensis* populations in the middle and lower reaches of the Yangtze River. The *D. similoides sinensis* distributed in the region had formed two clades, namely the Yangtze River clade and the Huai River clade. The *D. similoides sinensis* habitats in the middle and lower reaches of the Yangtze River have been fragmented because of the construction of dams and sluices since the 1950s. Habitat discontinuity and geographic segregation could accelerate regional genetic differentiation of *D. similoides sinensis* in the middle and lower reaches of the Yangtze River.

## CONFLICT OF INTEREST

None declared.

## AUTHORS' CONTRIBUTIONS

J.‐X.W., D.‐G.D., and Z.‐Z.Z conceived and designed the experimental plan. J.‐X.W., K.Z, W.‐P. Z. S.‐X.P, and X.‐X.X. performed the experiments. J.‐X.W. and Y.‐N.Z. analyzed and interpreted the sequence data. J.‐X.W. and D.‐G.D. drafted the manuscript. All authors have read and approved the final manuscript.

## Data Availability

The sequencing data of *Daphnia similoides sinensis *in this study were deposited in DRYAD (https://doi.org/10.5061/dryad.66p5487).
